# Neuroadaptive Training *via* fNIRS in Flight Simulators

**DOI:** 10.3389/fnrgo.2022.820523

**Published:** 2022-03-30

**Authors:** Jesse A. Mark, Amanda E. Kraft, Matthias D. Ziegler, Hasan Ayaz

**Affiliations:** ^1^School of Biomedical Engineering, Science, and Health Systems, Drexel University, Philadelphia, PA, United States; ^2^Advanced Technology Laboratories, Lockheed Martin, Arlington, VA, United States; ^3^Department of Psychological and Brain Sciences, College of Arts and Sciences, Drexel University, Philadelphia, PA, United States; ^4^Drexel Solutions Institute, Drexel University, Philadelphia, PA, United States; ^5^Department of Family and Community Health, University of Pennsylvania, Philadelphia, PA, United States; ^6^Center for Injury Research and Prevention, Children's Hospital of Philadelphia, Philadelphia, PA, United States

**Keywords:** neuroergonomics, fNIRS, neuroadaptive, prefrontal cortex, neurofeedback, learning, aviation, adaptive training

## Abstract

Training to master a new skill often takes a lot of time, effort, and financial resources, particularly when the desired skill is complex, time sensitive, or high pressure where lives may be at risk. Professions such as aircraft pilots, surgeons, and other mission-critical operators that fall under this umbrella require extensive domain-specific dedicated training to enable learners to meet real-world demands. In this study, we describe a novel neuroadaptive training protocol to enhance learning speed and efficiency using a neuroimaging-based cognitive workload measurement system in a flight simulator. We used functional near-infrared spectroscopy (fNIRS), which is a wearable, mobile, non-invasive neuroimaging modality that can capture localized hemodynamic response and has been used extensively to monitor the anterior prefrontal cortex to estimate cognitive workload. The training protocol included four sessions over 2 weeks and utilized realistic piloting tasks with up to nine levels of difficulty. Learners started at the lowest level and their progress adapted based on either behavioral performance and fNIRS measures combined (neuroadaptive) or performance measures alone (control). Participants in the neuroadaptive group were found to have significantly more efficient training, reaching higher levels of difficulty or significantly improved performance depending on the task, and showing consistent patterns of hemodynamic-derived workload in the dorsolateral prefrontal cortex. The results of this study suggest that a neuroadaptive personalized training protocol using non-invasive neuroimaging is able to enhance learning of new tasks. Finally, we outline here potential avenues for further optimization of this fNIRS based neuroadaptive training approach. As fNIRS mobile neuroimaging is becoming more practical and accessible, the approaches developed here can be applied in the real world in scale.

## Introduction

Training to learn complex skills, such as piloting an airliner or military plane, performing life-saving surgery in the hospital operating room, or being an emergency responder medic triaging and stabilizing patients in active warzone or accident scene, is difficult, time-consuming, expensive, and potentially dangerous to do. A common learning method is using high fidelity simulators under professional guidance, which may lead to mastery given enough time, but they do not guarantee *efficient* skill acquisition (Dotson et al., 2018). Because of individual differences, the speed of progression through each section of the training may vary. Moreover, accurate evaluation of when sufficient experience has been attained cannot be determined from just performance due to changes in strategy and effort (Metzger, 2001). Even as individuals display the same apparent performance, the amount of *mental effort* required by each may vary greatly (Debue and van de Leemput, 2014). This can result in higher-skilled learners being overtrained and wasting time and resources on unnecessary practice, or in lower-skilled learners proceeding while undertrained and failing at later stages that could have been avoided with more foundational practice. Therefore, there is an unmet need to measure the amount of effort required to achieve certain levels of performance and integrate that into training to make learning more efficient.

Increased skill is a result of training, and is generally defined as the accumulation of experience and the knowledge of how best to utilize it in achieving the desired goal. Skill can be acquired organically through experience without external direction, or it can be taught through specific training. However, a limitation of standardized testing is that it cannot measure effort, and a wide range of learners with differing proficiencies may be assessed to be at the same level. A possible remedy is to integrate cognitive workload measures into regular assessments (Ayaz et al., 2012b; Harrison et al., 2014). Absent these auxiliary measures of workload, it is very difficult to determine the effort gap between a learner of high skill vs. low skill. This information deficit persists for all types of assessments, from binary pass/fail to gradations of point awarding with letters or percent. This is because test performance is only an indirect measure of skill and confidence, which can be affected by external factors such as distraction (Dong et al., 2011). Nevertheless, the distinction between actual proficiency levels can be determined by analyzing mental workload during task performance (Bunce et al., 2011). Workload and performance can be continuously monitored during training and used to dynamically alter task difficulty to maximize engagement and learning, which supplements standard post-training assessment (Ayaz et al., 2013).

Personalizing training methods to an individual operator assists in learning complex tasks in an efficient manner. The process of learning and mastering a practical skill is different for each person, but typically follows an established path of aptitude development (Vygotsky, 1978; Ebbinghaus, 2013). The standard measures of proficiency are *in situ* performance and behavior; however, by taking cognitive workload into account, a more accurate and objective measure of mastery can be developed (Bunce et al., 2011; Ayaz et al., 2013). Workload can be measured in several direct and indirect ways including primary task performance, secondary task performance, subjective surveys, physiological measures such as skin conductance and heart rate variability, and neurological measures including electroencephalogram (EEG) and functional near-infrared spectroscopy (fNIRS) (Scerbo et al., 2001; John et al., 2002; Hart, 2006; Wilson and Russell, 2007; Durantin et al., 2014; Mandrick et al., 2016; McKendrick et al., 2016; Zander et al., 2016; Ayaz and Dehais, 2019). Primary task performance measures will differ depending on the experiment or training, but always rely exclusively on objective and unambiguous assessments. By incorporating neuroimaging correlates of workload into the process, training for complex real-world tasks can be improved.

To successfully integrate all of the available information from a learner engaged in a lesson, we need a solid foundational understanding of how internal and external measures interact. The relationship between behavior and mental effort can be modeled by an inverted U-shaped curve of performance vs. arousal known as the Yerkes-Dodson law (Sibi et al., 2016). This graph was originally described in terms of physical adaptability vs. stress (Hancock and Chignell, 1986), but the concept has been shown to be widely applicable to non-physical tasks and mental effort as well (Sibi et al., 2016). In this model, given a task of constant difficulty, a learner of low skill attempting to the best of their ability to succeed will experience high workload but be unable to achieve high performance, an indicator of low efficiency (Ayaz et al., 2012a). As skill increases, workload descends to a manageable level and performance converges toward a theoretical maximum. However, after a certain point when the relative skill to difficulty ratio is too high, the learner may become bored and begin to disengage with the task, leading to decreased performance. This has been demonstrated in multiple ways including car driving studies, in which more experience can lead to distraction and accidents (Paxion et al., 2014; Solovey et al., 2014). This workload-performance relationship concept can be applied during adaptive training to dynamically alter task difficulty and maintain learners at peak efficiency. At this peak point when performance is maximized and workload is neither too high nor too low, the efficiency of skill acquisition increases, particularly in the case of inducing a state of mental flow (Afergan et al., 2014).

As practice continues and skill increases, several different patterns in brain activation can be seen based on location, task type, and length of training (Kelly and Garavan, 2005). Localized and connected brain regions may experience one of four types of changes: (1) an increase in activity; (2) a decrease in activity; (3) a redistribution of activity, in which certain areas are more active at the start of learning and some are more active at the end, but all are involved in the overall process; (4) a reorganization of activity, in which wholly separate brain regions are used at the start and end of training, often associated with developing new strategies as expertise is gained (Ayaz and Dehais, 2021). The prefrontal cortex (PFC) is thought to be a key constituent of attention and high-level executive control, and the majority of studies examining task practice observed activation changes in this area with ongoing practice (Kelly and Garavan, 2005; Ayaz et al., 2012b).

Autonomic physiological signals can provide useful metrics to inform workload measurements. For instance, eye tracking is used in a variety of fields ranging from psychology to human computer interaction to neuromarketing (Duchowski, 2002). Remote eye tracking is non-invasive, not distracting to learners, and easy to set up and calibrate on most monitors. Eye movements can be analyzed by scan speed, saccade velocity and frequency, fixation count and duration, and combinations of the above, in addition to pupil diameter changes (Goldberg and Kotval, 1999; Jacob and Karn, 2003; Ahlstrom and Friedman-Berg, 2006). This provides information on attentional distribution and the ergonomics of interface design on top of workload correlates. Another relevant autonomous signal is heart rate and heart rate variability. The interaction of the sympathetic and parasympathetic nervous systems relayed by low- and high-frequency variability provides insight on workload and task performance (Durantin et al., 2014). One method of validating these measures that exist outside of learners' perception is subjective workload surveys such as the NASA task load index (NASA-TLX) (Hart, 2006). These are excellent at measuring perceived effort in multiple dimensions, but are difficult to compare across subjects who may have different internal models and ranges of answers. By combining these physiological measures, along with neuroimaging described below, it is possible to paint a richer picture of mental state.

Neuroimaging can be used to more accurately and objectively read moment-to-moment cognitive workload for both instantaneous and overall measures. Neuroergonomic approaches based on measures of human brain hemodynamic or electromagnetic activity can provide for sensitive and reliable assessment of mental workload in complex work environments (Parasuraman, 2011). Functional near-infrared spectroscopy (fNIRS) is a lightweight, wearable, and portable brain imaging modality that can derive correlates of mental workload for practical neuroergonomic purposes (Curtin and Ayaz, 2018; Ayaz et al., 2019). It accomplishes this *via* the calculation of oxygenated and deoxygenated blood in the cortex using the modified Beer-Lambert law using two wavelengths of light in the optical window of tissue, in which hemoglobin has a higher absorption than water (Villringer and Chance, 1997; Izzetoglu et al., 2005). fNIRS has been used to measure workload in a variety of complex tasks such as performing surgery, driving, flying, and coordinating air traffic control (Ayaz et al., 2012b; Harrivel et al., 2013; Naseer and Hong, 2015; Foy et al., 2016; Gateau et al., 2018; Singh et al., 2018; Causse et al., 2019). Because data is recorded continuously and can be analyzed both online and offline in real world situations, fNIRS is ideal for adaptive training done in real time.

In this study we aimed to develop and assess a novel neuroadaptive training approach using a desktop flight simulator and wearable fNIRS neuroimaging. We utilized three distinct complex and realistic tasks performed during four sessions spread over 2 weeks of time. Subjects were randomly assigned to one of two conditions: the neuroadaptive group, who progressed based on both performance and mental workload measures; and the control group, who progressed based solely on task performance. We analyzed the effect of neurofeedback (received in the form of task difficulty adjustment as described in the next section) within an adaptive training protocol on speed of progression, ability to retain and apply new skills, and overall workload levels.

## Methods

### Participants

Twelve participants (4 female) between the ages of 20 and 28 (age 24.9 ± 2.9 years) volunteered for the four session, two-week long study protocol. All confirmed that they met the eligibility requirements of being right-handed *via* the Edinburgh Handedness Inventory, had vision correctable to 20/20, did not have a history of brain injury or psychological disorder, and were not on medication affecting brain activity. Prior to the study all participants signed written informed consent forms approved by the Institutional Review Board of Drexel University. Participants were given monetary compensation for their time.

### Recording

Functional near-infrared spectroscopy was recorded using an Imager Model 1100 by fNIR Devices, LLC (Potomac, MD). This device records at 16 optode locations over the prefrontal cortex using four light emitting diodes (LED) as sources and 10 detectors at a rate of 2 Hz. COBI Studio software recorded light intensity data with three channels for each optode: 730 and 850 nm wavelengths and ambient light (Ayaz et al., 2011). The sensors were aligned with the centerline of the head and placed above the brow for repeatable measurements (Ayaz et al., 2011).

### Surveys

The NASA Task Load Index (NASA-TLX) was used to measure subjective workload, and was given once per task at the end of each block, for a total of three per session (12 total). This is a questionnaire of six aspects of mental workload, each of which is graded on a 21-point Likert scale. Learners self-rate on ranges of mental, physical, and temporal demand, as well as effort, performance, and frustration. These can be combined into one overall subjective workload measurement that can be compared with other results.

### Experiment

#### Setup

**Figure 1:**
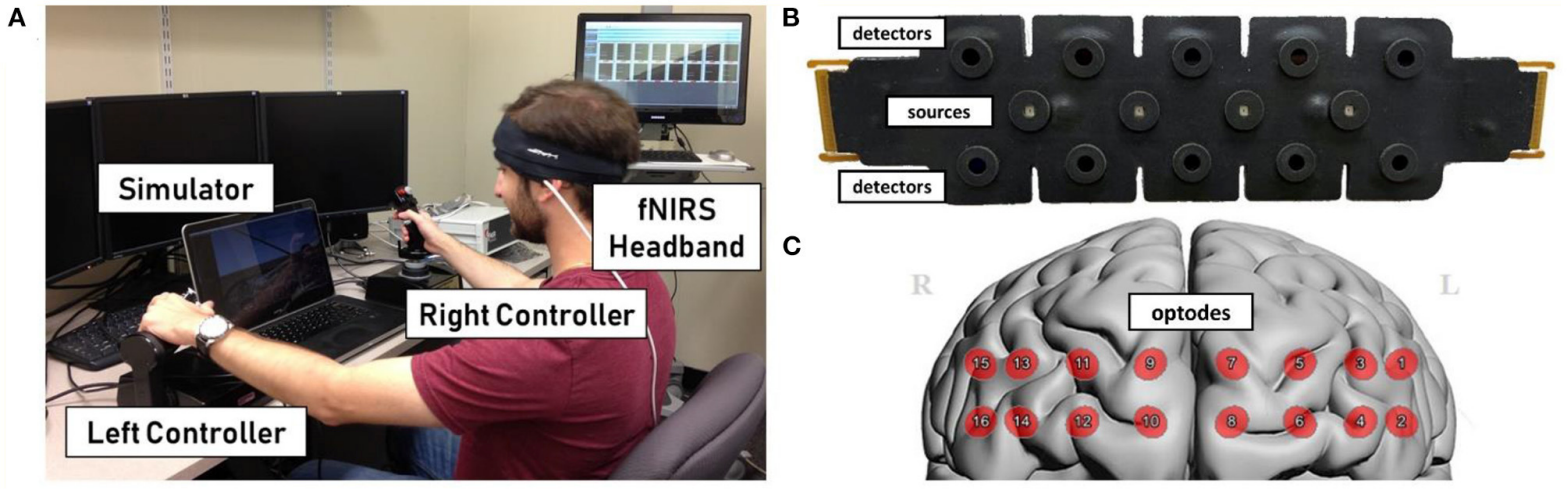
**(A)** Example of participant during experimental setup. Participants were seated at a desktop computer during actual task and fitted with fNIRS on the head. **(B)** Ultra-thin flexible sensor pad with 10 light detectors and 4 LED light sources. **(C)** Optode layout from sensor superimposed on model of the cortex.

The experiment was conducted over four 1-h sessions spaced out over 2 weeks. Participants sat in a room free from distraction at a comfortable distance from a computer monitor, and performed the tasks using a standard mouse plus two Thrustmaster HOTAS controllers, a joystick and throttle (Figure 1). All tasks were presented using the flight simulator software Prepar3D^®^ developed by Lockheed Martin. The three tasks described below were based on our previous studies (Ayaz et al., 2012b; Choe et al., 2016) and presented in pseudo-randomized block order, balanced between participants. Each included one reference trial given at the lowest difficulty level, plus four training trials that adapted between sessions based on participant condition. The fourth and final session presented three training trials plus two transfer trials at the maximum difficulty for each task. Each trial took ~120 s, and each task block averaged 15 min total.

#### Landing Task

**Figure 2:**
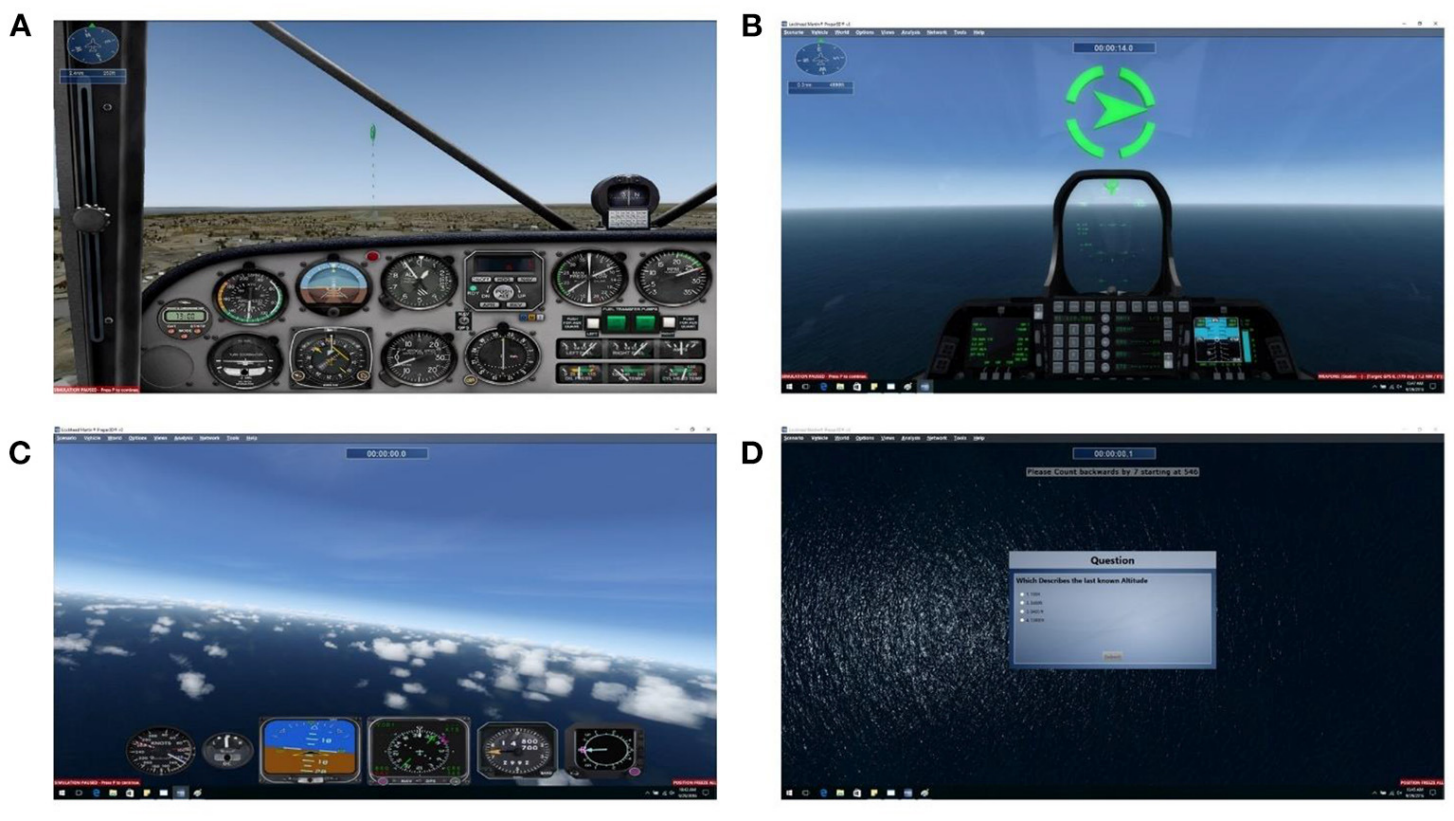
Sample screens displaying the three tasks used. **(A)** Landing task viewpoint. **(B)** Rings task viewpoint. **(C)** Situation awareness task video. **(D)** Situation awareness task question screen.

The goal of this task was to use both the joystick and throttle controllers to land a plane that begins midair on a runway ahead of the starting position (Figure 2A). Low difficulties limit plane control to roll only and have set autopilot for speed and pitch. As difficulty increases through the eight available levels, more control is progressively given to the participant, and conditions such as landing below certain airspeeds and vertical speeds are given to succeed. In addition, harder difficulties obscured vision with fog or rain, and included high winds that caused turbulence. Performance was calculated by the smoothness of flying, calculated by the difference between the deviation of actual flight path from the root mean square calculated ideal path.

#### Ring Task

The goal of this task was to use the joystick controller to fly a plane through several rings suspended in midair (Figure 2B). At the lowest level, the rings were placed in a straight line and remained stationary. As difficulty increased through the nine available levels, the rings moved laterally back and forth at increasing speeds. Performance was calculated by ratio of rings flown through and the stability of flight through each ring as calculated by the roll and pitch of the plane as it flew through the rings.

#### Situation Awareness Task

The goal of this task was to maintain awareness of the gauges on the dashboard of a virtual plane and then recall their values after a distraction period. Participants viewed 90 s prerecorded first-person pilot-perspective videos of planes in flight, after which they were required to do mental subtraction for 15 s to prevent immediate recall (Figure 2C). Participants were then asked a series of multiple-choice questions regarding the plane's airspeed, heading, altitude, and so on (Figure 2D). The seven difficulty levels were modulated by the smoothness of the flight, thereby changing the values more or less dramatically, and the number of gauges to recall (between three and eight). Performance measures were calculated based on percentage of correct answers.

### fNIRS Signal Processing

Light intensity values were processed using a lowpass Finite Impulse Response (FIR) filter with cutoff 0.1 Hz to attenuate the high frequency noise, respiration, and cardiac cycle effects and a Sliding-window Motion Artifact Rejection (SMAR) algorithm for removing motion artifacts and potential saturations (Ayaz et al., 2010). fNIRS data for each training block were extracted using time synchronization markers received through serial port during experiment and hemodynamic changes for each of 16 optodes during each trial block were calculated separately using the Modified Beer Lambert Law (MBLL). The hemodynamic response at each optode was baselined to the start of each block and averaged across time for each task trial to provide a mean hemodynamic response at each optode for each block. The final output of each optode was oxygenated hemoglobin (HbO) as the main biomarker.

Linear regression was used to calculate the rate of change of hemoglobin concentration values over 90 s for each trial using the slope method of determining workload (Mandrick et al., 2013). This method fits the data from the start to the end of each block, with higher magnitudes of the slope value indicating stronger responses. Learning-induced workload correlates were measured from optodes covering the left lateral prefrontal cortex, which has been associated with skill-related changes in the brain (Ayaz et al., 2013). For the first three sessions, each of the four training trials within each task block were compared over time to determine if workload was increasing, decreasing, or remaining the same. This was used for the neuroadaptive group in difficulty adjustment as described below.

### Adaption Decision Tree

**Figure 3:**
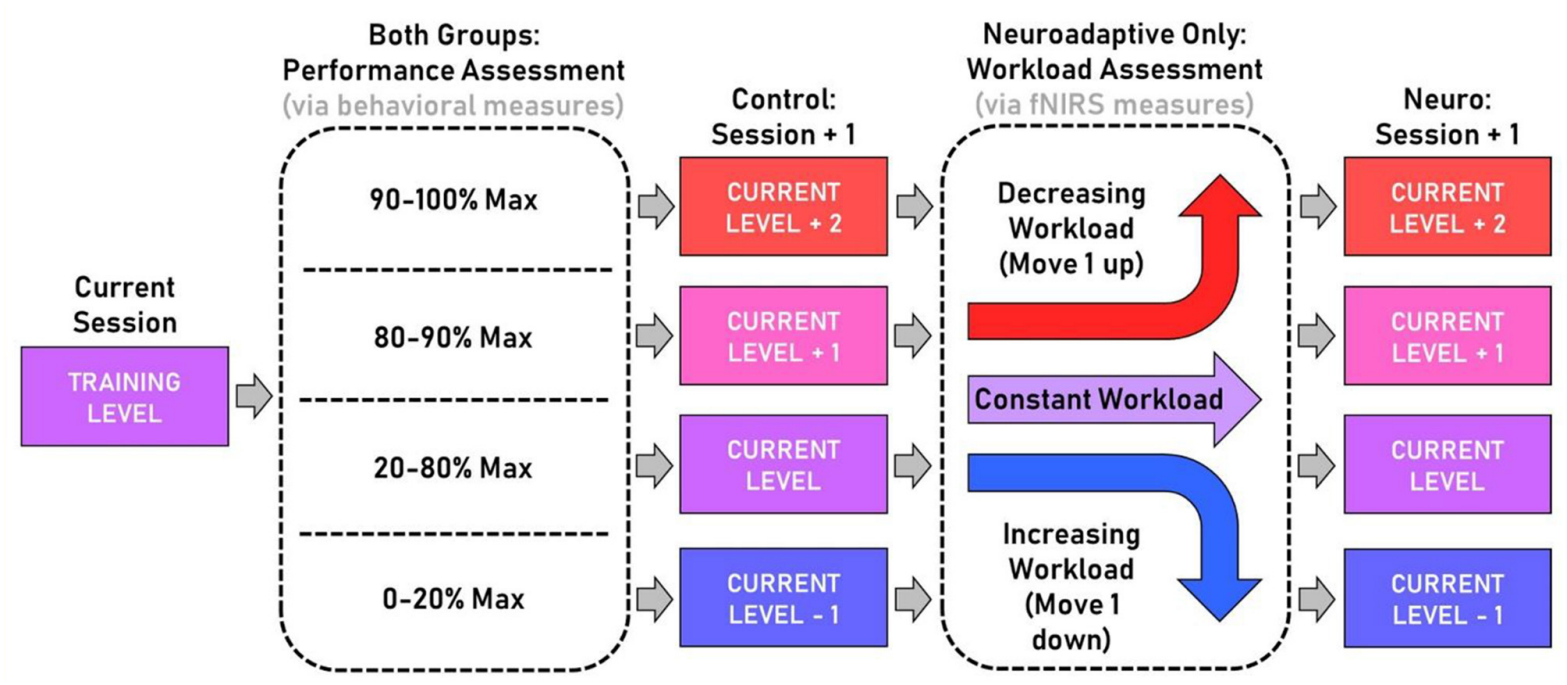
Flowchart of the adaptive training protocol. All participants began training tasks at level 2 during the first session. Then the performance assessment was used to sort participants in both groups into four potential level changes during the next session. Only the neuroadaptive group got the workload assessment adjustment, which modified their next training level up one, down one, or not at all based on mental workload changes across the session.

Session-based feedback was utilized for each task to update the respective task difficulty levels for the upcoming session. For each task within a session, participants were first classified into one of four groups based on their performance scores: below 20% theoretical maximum score, 20–80% max, 80–90% max, and 90–100% max. These classifications were used to adjust the difficulty levels for the following session. For the control group, only the sum of performance scores dictated the following training level for the next session (Session + 1). For the neuroadaptive group, one additional step was used following the performance classification. Based on the slope method processing as described above to classify learning workload into increasing, decreasing, or neutral states, further level adjustment could occur. Increasing workload indicating too much required effort lowered the following level by one, decreasing workload indicating too little effort for performance increased the following level by one, and neutral or unchanging workload resulted in the same proceeding level as the control group. For this study, we capped the increase at +2 and decrease at −1, to have a balanced min and max adaptation across both groups. Figure 3 outlines the algorithm used.

The difficulty level of each task remained the same within a session. All behavioral and neuroimaging data processing was conducted after the completion of each session to inform the next session, and output was used as described in Figure 3 to select the following session's parameters for each task for the respective participants.

### Statistical Analysis

Linear mixed models were used for statistical analysis of behavioral, subjective, and fNIRS measures. Separate models for each behavioral measure and fNIRS optode were used and included *group* and *session* fixed factors, *subject* as random factor, and *level* as covariate. The model fixed terms were *group* + *session* + group^*^session + *level* + group^*^level and with random term of *subject*. *Post-hoc* comparisons were performed for all pairs of factor levels and multiple comparisons were corrected with Bonferroni method. All model figures used Standard Error of the Mean (SEM) as whiskers.

## Results

The results below present training level progress, perceived effort (NASA TLX workload survey), behavioral performance (task specific variables), and fNIRS workload measures (oxygenated hemoglobin changes) for each participant group and each task.

### Landing Task

#### Performance and Subjective Measures

**Figure 4:**
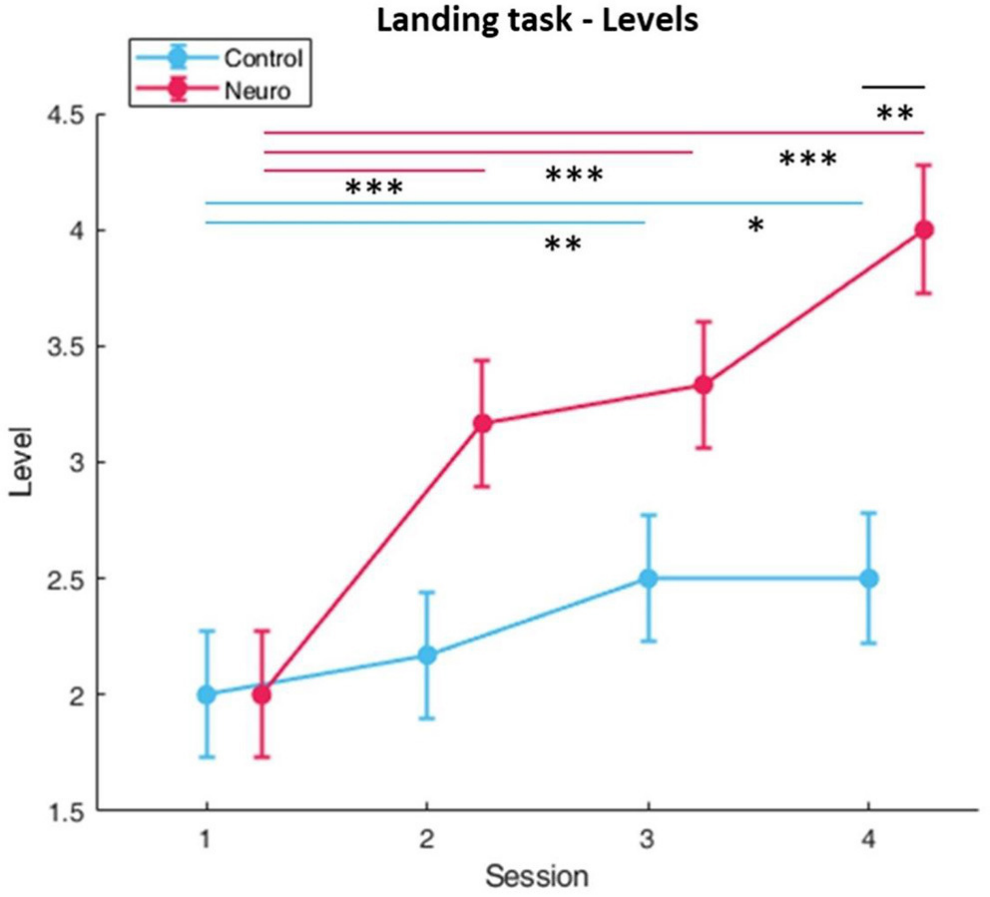
Training levels reached for landing task over each session per experimental group. Neuroadaptive group reached significantly higher levels (whiskers are SEM, *p < 0.05, **p < 0.01, ***p < 0.001).

**Figure 5:**
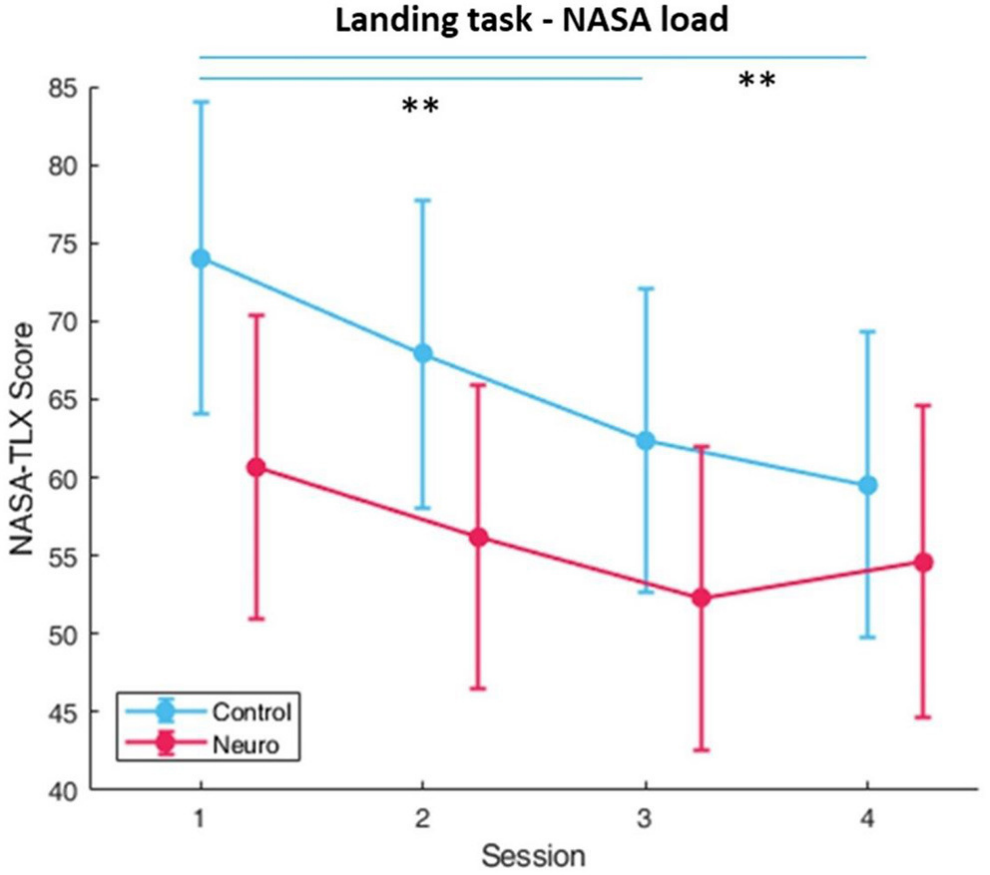
Subjective workload calculated by sum of NASA-TLX. Significant condition by level differences were found (whiskers are SEM, **p < 0.01).

**Figure 6:**
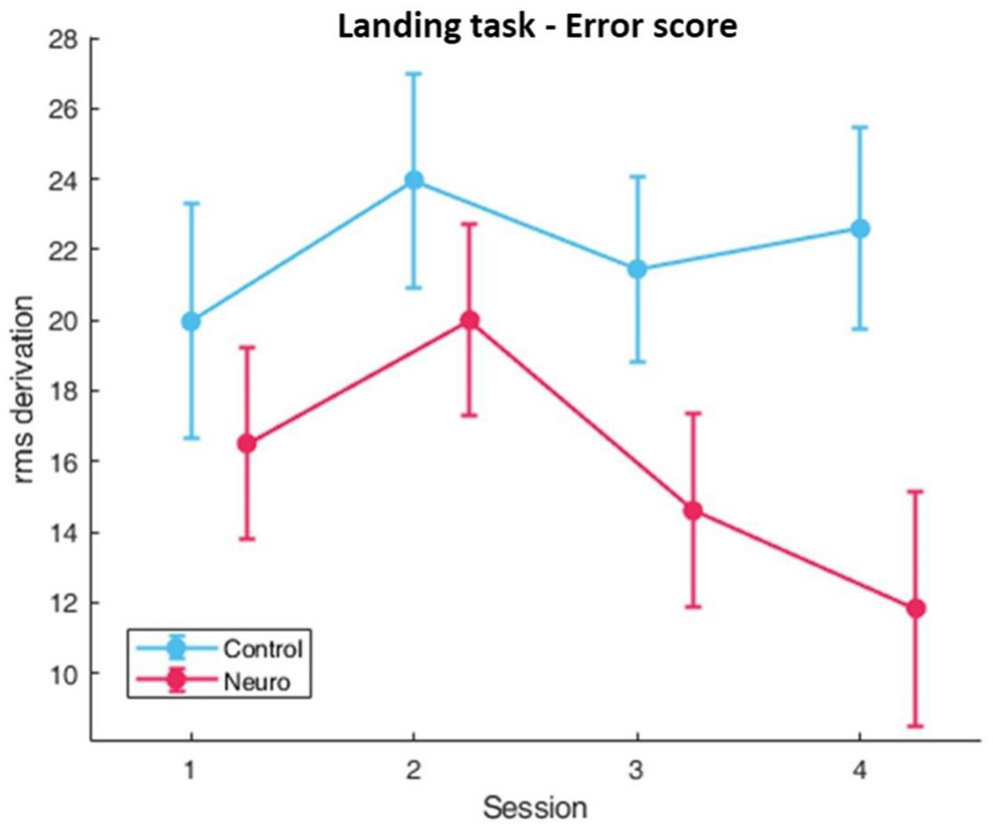
Behavioral performance (error) as measured by flight stability, the root mean square difference between actual and ideal flight path for Landing Task. Lower values indicate better performance. Significant condition by level interaction was found (whiskers are SEM).

Participants in the neuroadaptive group reached higher difficulty levels during the 2-week training period than the control group, with significant differences in group [*F*_(1, 10)_ = 5.35, *p* < 0.05], session [*F*_(3, 161)_ = 42.4, *p* < 0.001], and group by session interaction [*F*_(3, 161)_ = 14.5, *p* < 0.05] as depicted in Figure 4. In addition, NASA-TLX self-reported workload measures (Figure 5) had significant group and level interaction [*F*_(1, 162.5)_ = 9.65, *p* < 0.01] with neuroadaptive group reporting slightly lower workload throughout the training sessions. Flight stability measures as calculated by the root mean squared deviation from an ideal flight path, with the minimal amount of jerks in three dimensional acceleration changes, showed group by level interaction differences [*F*_(1, 124.2)_ = 6.51, *p* < 0.001] shown in Figure 6.

#### fNIRS Measures

**Figure 7:**
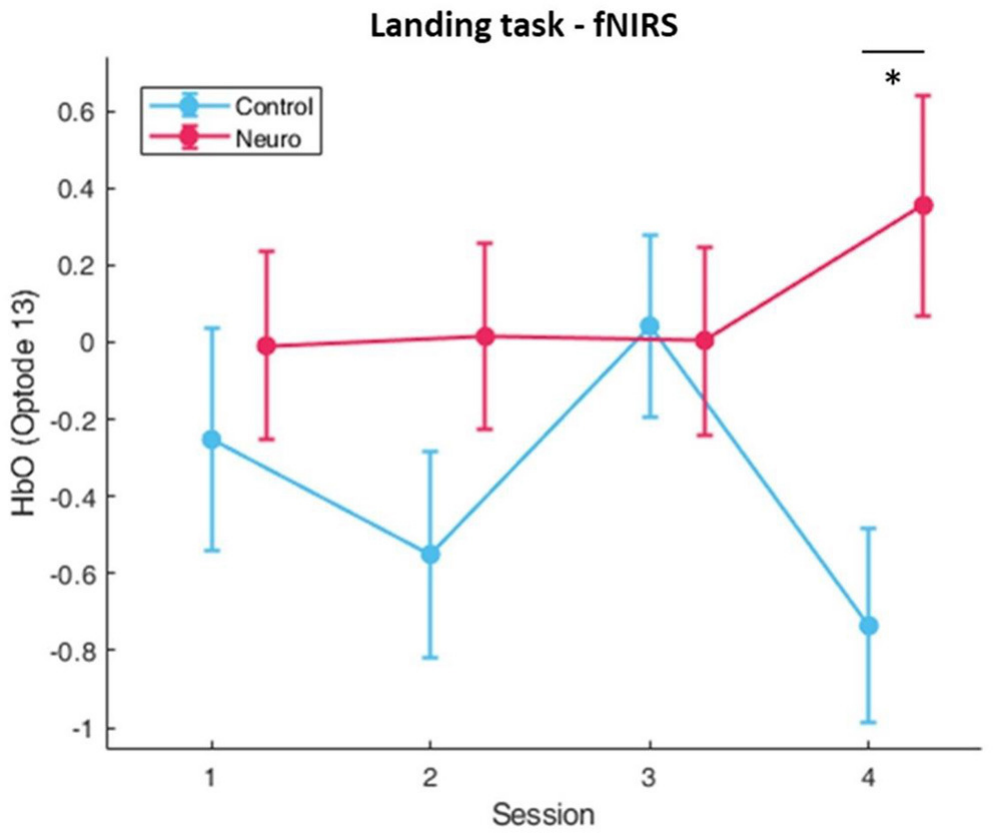
Oxygenated hemoglobin changes over time corrected with level covariate. Significant condition and session interactions were found (whiskers are SEM, *p < 0.05).

Significant factors and interactions were found for several optodes in the landing task. For main effect of session, the most significant optodes were optode 8 [*F*_(3, 163.8_ = 4.07, *p* < 0.01] in the medial prefrontal and optode 16 [*F*_(3, 163.7)_ = 4.29, *p* < 0.01] in the right dorsolateral PFC. Right dorsolateral prefrontal cortex areas also showed significant interactions between group and session in optode 13 [*F*_(3, 163.7)_ = 4.29, *p* < 0.01], and moreover *post-hoc* comparisons at this optode also showed a significant difference between groups at session 4 [*F*_(1, 27.3)_ = 8.18, *p* < 0.05] (Figure 7). Directly below this location, optode 14 also had significant interaction [*F*_(3, 163.7)_ = 3.80, *p* < 0.05]. The full list with all main effects and interactions for all optodes are in Supplementary Tables S1–S3.

### Situational Awareness Task

#### Performance and Subjective Measures

**Figure 8:**
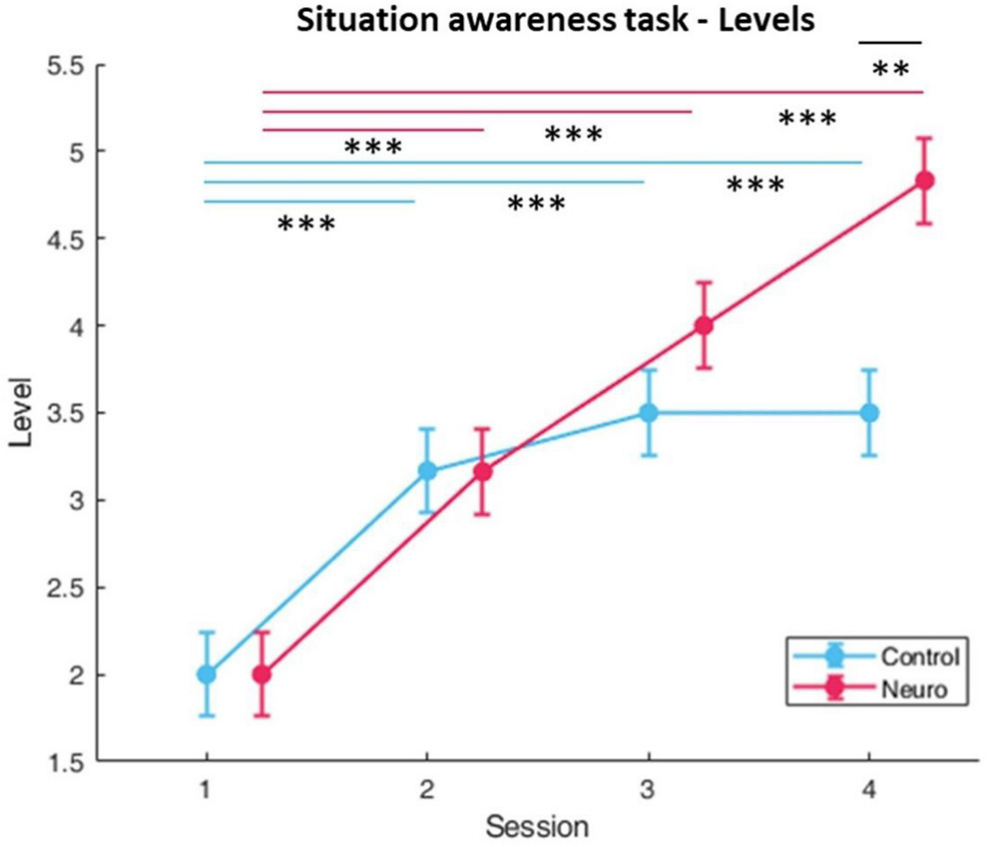
Training levels reached for situational awareness over each session per experimental group. Neuroadaptive group reached significantly higher levels in session 4 (whiskers are SEM, **p < 0.01, ***p < 0.001).

**Figure 9:**
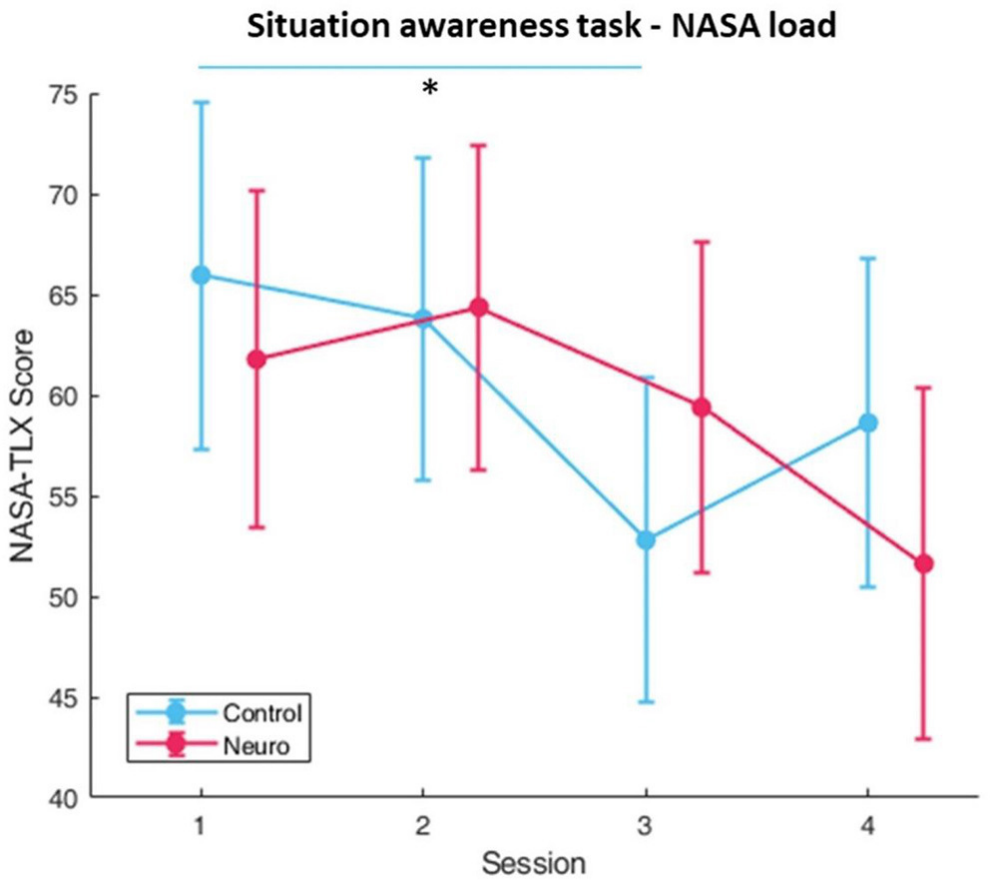
Subjective workload calculated by sum of NASA-TLX self-reports. A significant interaction between condition and session was found (whiskers are SEM, *p < 0.05).

**Figure 10:**
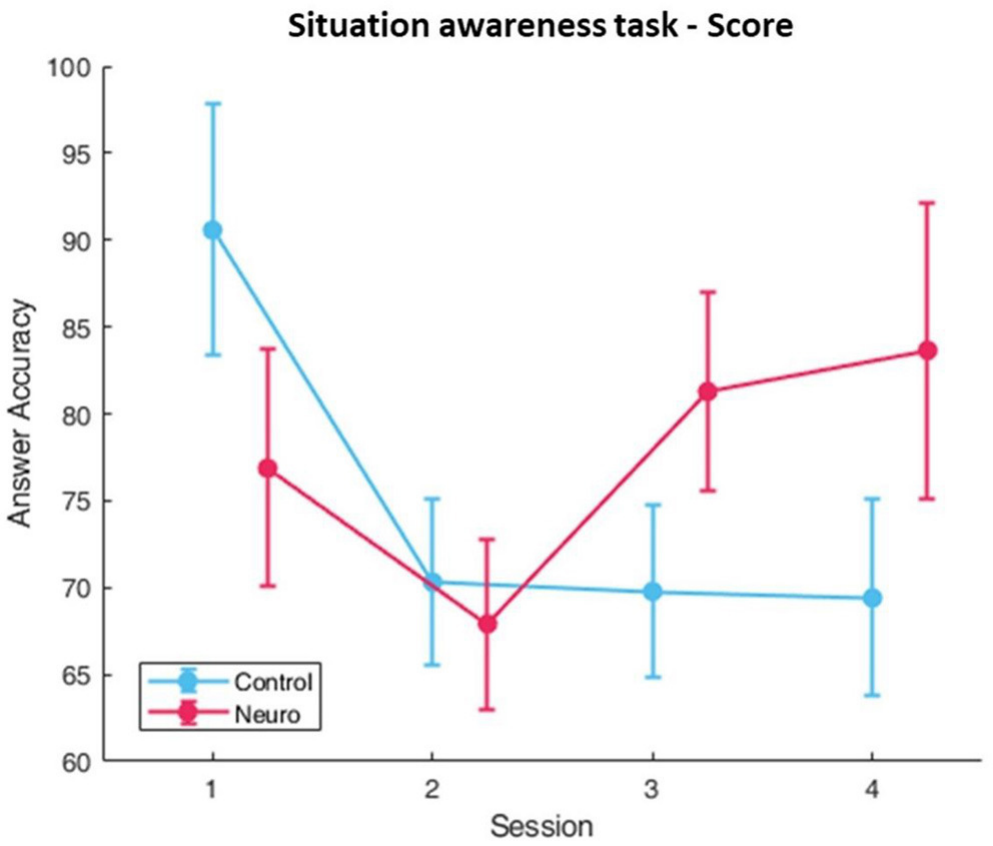
Behavioral performance as measured by the ratio of correct answers. No significant differences or interactions were found (whiskers are SEM).

Participants in the neuroadaptive group consistently progressed to higher difficulty levels, whereas the control group plateaued (Figure 8), with a significant interaction between group and session [*F*_(3, 161)_ = 19.0, *p* < 0.001]. *Post-hoc* analysis showed significant increases over time for both, with a group difference found for level in session 4 [*F*_(1, 13.8)_ = 14.6, *p* < 0.01]. Subjective workload decreased over time for both groups over sessions [*F*_(3, 160.8)_ = 5.57, *p* < 0.01], with a significant group and session interaction [*F*_(3, 160.8)_ = 4.17, *p* < 0.01] (Figure 9). The behavioral performance as measured by proportion of questions answered correctly did not show any significant differences or interactions (Figure 10).

#### fNIRS Measures

**Figure 11:**
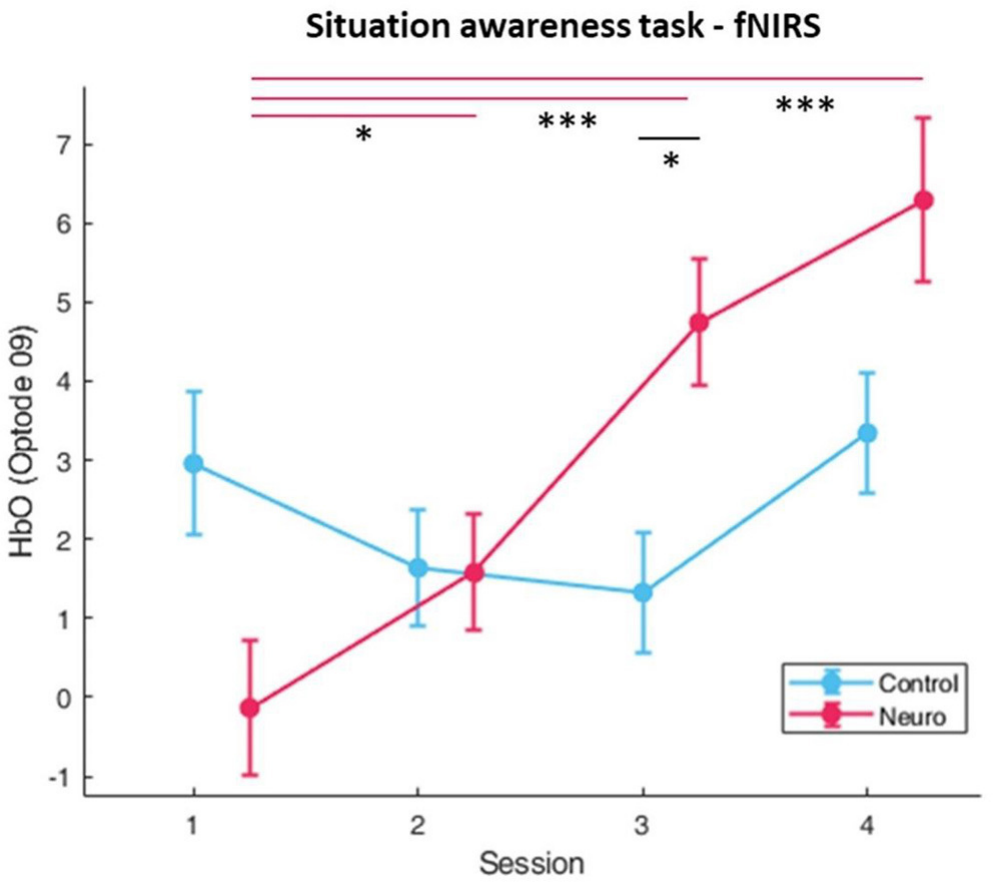
fNIRS cognitive workload correlates as measured by oxygenated hemoglobin (HbO) in optode 4, the left prefrontal cortex, in the situational awareness task. Significant differences and interaction between group and session was found. Neuroadaptive group also showed higher HbO in session 3 (whiskers are SEM, *p < 0.05, ***p < 0.001).

Significant differences in main factor of group were found in the right medial PFC in optode 9 [*F*_(1, 82.9)_ = 9.30, *p* < 0.01] and optode 10 [*F*_(1, 87.4)_ = 8.09, *p* < 0.01]. The fNIRS measure was found to be highly sensitive to differences between session across the prefrontal cortex, and the most significant location was in the left medial prefrontal cortex in optode 5 [*F*_(3, 159.7)_ = 17.4, *p* < 0.001]. The prefrontal cortex was found to be widely sensitive to group and session interaction (Figure 11), with the most significant optodes being optode 9 [*F*_(3, 155.9)_ = 11.1, *p* < 0.001] and optode 15 [*F*_(3, 160.4)_ = 10.9, *p* < 0.001], with optode 15 having *post-hoc* significant differences between groups in session 3 [*F*_(1, 16.8)_ = 11.7, *p* < 0.05]. Finally, the right medial PFC was found to be sensitive to group and level interaction in optode 09 [*F*_(1, 158)_ = 9.40, *p* < 0.01] and optode 10 [*F*_(1, 146)_ = 7.75, *p* < 0.01]. The full list with all main effects and interactions for all optodes are in Supplementary Tables S1–S3.

### Ring Task

#### Performance and Subjective Measures

**Figure 12:**
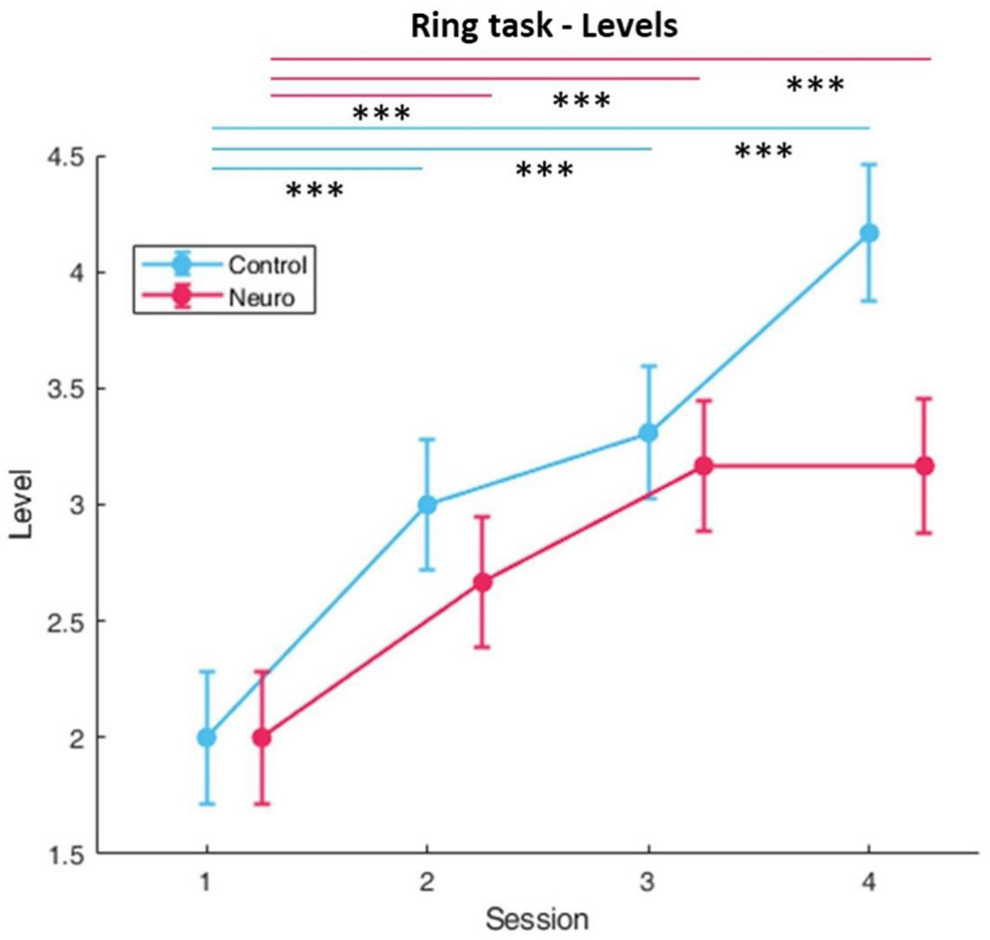
Training levels reached for ring task over each session per experimental group. Both conditions displayed significant level increases over time (whiskers are SEM, ***p < 0.001).

**Figure 13:**
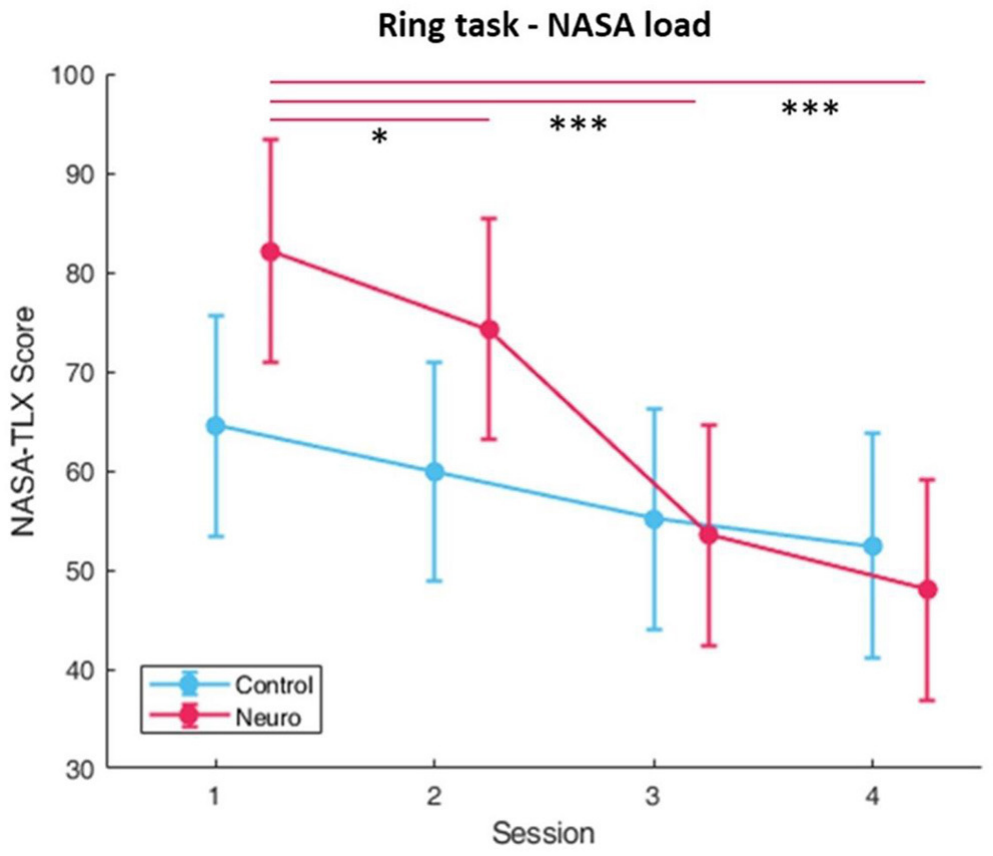
Subjective workload calculated by sum of NASA-TLX self-reports for rings task. A significant condition by session interaction was found, and only the neuroadaptive group had significant decreases in self-reported workload (whiskers are SEM, *p < 0.05, ***p < 0.001).

**Figure 14:**
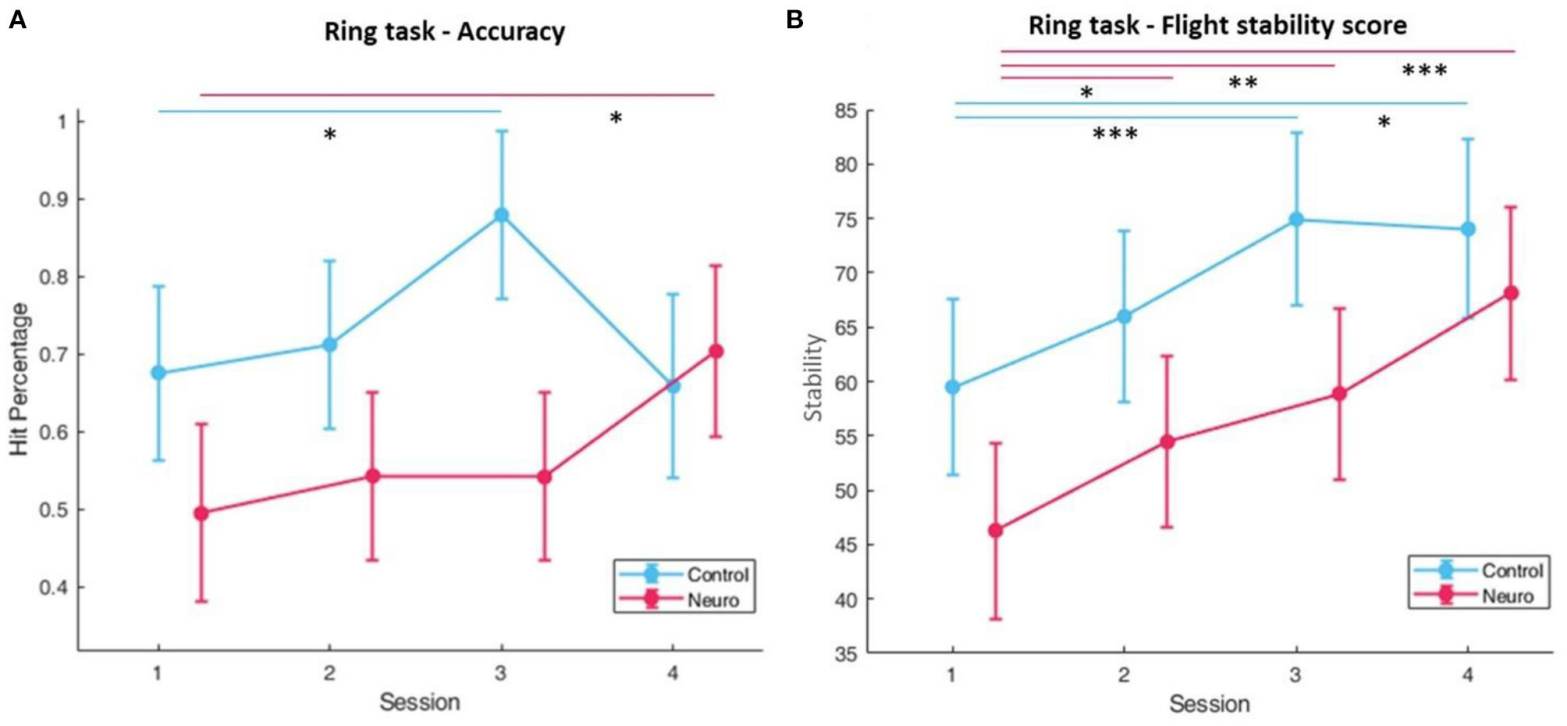
**(A)** Behavioral performance as measured by the accuracy, or ratio of rings successfully flown through for the ring task. A significant interaction between condition and session was found. **(B)** Behavioral performance calculated by plane orientation through rings. Both conditions significantly improved over time, but only neuroadaptive maintained that increase throughout all sessions (whiskers are SEM, *p < 0.05, **p < 0.01, ***p < 0.001).

Level progression in both groups consistently increased over the first three sessions, and in session four the control group reached slightly higher difficulty levels than the neuroadaptive group (Figure 12). There was a significant interaction between group and session [*F*_(3, 161)_ = 6.34, *p* < 0.001]. Both groups showed decreases in subjective workload over time (Figure 13), with a significant interaction between group and session [*F*_(3, 157.6)_ = 8.82, *p* < 0.001], with *post-hoc* decreases in NASA-TLX scores only found in the neuroadaptive group. For behavioral performance measures, the ratio of rings flown through per trial (which varied due to participant skill and time limit) had a significant interaction between group and session [*F*_(3, 159.4)_ = 6.53, *p* < 0.001], with the control group improving in session 3 but falling after, and neuroadaptive group improving in session 4 (both significant at *p* < 0.05) (Figure 14A). For the flight stability measures, calculated by plane orientation through rings, both groups showed an increase in performance over time with a significant difference between sessions [*F*_(3, 158.1)_ = 14.1, *p* < 0.001], and *post-hoc* calculations showed a more consistent increase in the neuroadaptive group (Figure 14B).

#### fNIRS Measures

**Figure 15:**
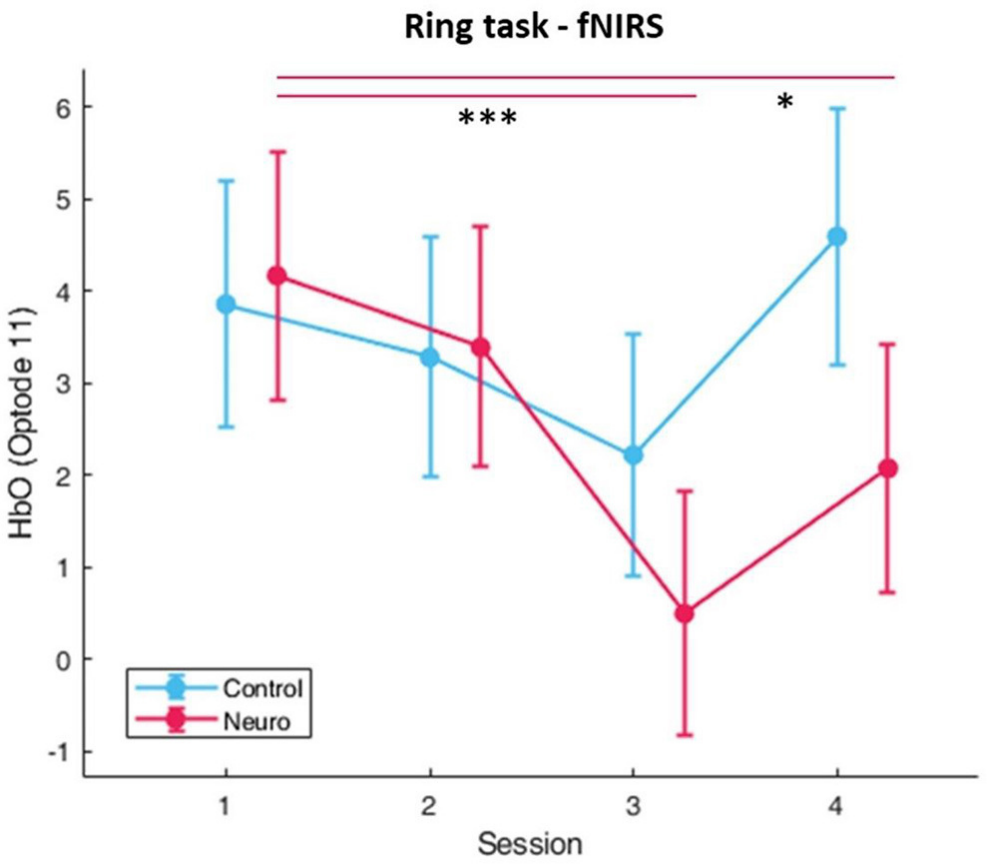
Oxygenated hemoglobin changes over time corrected with level covariate. Significance found between groups, session, and interaction, as well as condition and level interaction. (Whiskers are SEM, *p < 0.05, ***p < 0.001).

The right medial prefrontal cortex was found to be highly sensitive to the main effect of group. From most to least significant, optode 11 [*F*_(1, 26.1)_ = 10.3, *p* < 0.01], optode 12 [*F*_(1, 28.7)_ = 7.96, *p* < 0.01], optode 10 [*F*_(1, 37.6)_ = 6.81, *p* < 0.05], and optode 9 [*F*_(1, 32.6)_ = 6.64, *p* < 0.05]. The fNIRS measure was found to be highly sensitive to differences between session across the prefrontal cortex, and the most significant location was in the right medial prefrontal cortex in optode 11 [*F*_(3, 150.6)_ = 14.5, *p* < 0.001] (Figure 15). Group and session interaction was most significant in optode 11 [*F*_(3, 150.6)_ = 3.34, *p* < 0.05] in the right medial PFC and also optode 3 [*F*_(1, 159.4)_ = 3.28, *p* < 0.05]. Finally, the right medial PFC was found to be sensitive to group and level interaction in optode 9 [*F*_(1, 161.3)_ = 12.0, *p* < 0.001], optode 10 [*F*_(1, 158.8)_ = 11.0, *p* < 0.01], optode 11 [*F*_(1, 156.3)_ = 19.9, *p* < 0.001], and optode 12 [*F*_(1, 146)_ = 14.3, *p* < 0.001]. The full list with all main effects and interactions for all optodes are in Supplementary Tables S1–S3.

## Discussion

In this study, we captured wearable neuroimaging-based mental workload during flight simulator task practice over 2 weeks, and utilized that for a new adaptive training approach that is based on brain measures.

The training protocol included four sessions and utilized realistic piloting tasks with up to nine levels of difficulty. Learners started at the lowest level and their progress adapted based on either behavioral performance and fNIRS measures combined (neuroadaptive) or performance measures alone (control).

The main contribution of this study was the development and evaluation of this novel neuroadaptive training approach that adjusted subsequent training task difficulty level based on mental workload and behavioral performance on the current task. We developed a two-tiered system of task level adjustment (Figure 3) where participants were individually sorted first by performance, allowing for a range of next session options ranging from remaining at the same level, decreasing one level, increasing one level, or increasing two levels. This was then modulated by fNIRS-measured mental workload in the left dorsolateral prefrontal cortex, which is associated with learning. Increasing workload over trials adjusted the level progression down, and decreasing workload adjusted it up. This algorithm was intended to provide a generalizable framework that could be applied to a range of different applications.

We used the changes in oxygenated hemoglobin of the left dorsolateral prefrontal cortex to determine mental effort during tasks and estimate internal skill development based on previous longitudinal studies with verbal and spatial working memory and similar aviation tasks (Bunce et al., 2011; Ayaz et al., 2012a, 2013; McKendrick et al., 2014). While this area of the brain did show some relevant response to each task to assist in the personalized difficulty progression, other areas may be more sensitive to specific task-related mental workload. It may even be more effective to apply a wider bilateral or medial range of the prefrontal cortex to analyze a novel task without any prior knowledge. In the future, by using the results of this study, we will be able to better isolate specific optodes that correlate with skill acquisition over trials within a single session for different tasks.

### Findings and Insights

Overall, participants in the neuroadaptive group were found to have significantly more efficient training, reaching higher levels of difficulty in the landing and situation awareness tasks, preventing advancement beyond skill level in the ring task, improving performance over the full training period, and showing consistent patterns of hemodynamic-derived workload in the dorsolateral prefrontal cortex. In the following section, we describe specific patterns across the three tasks individually, which display two types of patterns: one for the landing and situation awareness tasks, and one for the ring task.

In the landing task, neuroadaptive participants were able to reach higher difficulty levels, with the greatest increase being seen from session one to session two (Figure 4). This occurred because all participants were able to achieve at least proficient performance, but only the neuroadaptive group jumped two difficulty levels due to their measured low workload, which may suggest that our designed task is too simple at the beginning, and may benefit from added complexity. *Post-hoc* tests also reveal that by session 4, the neuroadaptive group reached significantly higher training levels, in part due to more improvement during session 3.

Behavioral performance was measured by flight stability, specifically the ability to maintain a consistent trajectory during the descent to runway, as well as keep the plane from jerking during wind in higher difficulty levels (Figure 6). Both groups display a slight increase in calculated rms difference from an ideal path in session two, representing worse performance, but only the neuroadaptive group showed an improvement in performance over time. This is notable due to the higher training levels achieved by these participants. In addition, a significant interaction between group and level was found, showing that neuroadaptive participants also performed better than control participants while at the same training level.

Significant interactions between group (control vs. neuroadaptive) and session (4 training sessions) were found for two optodes in the right DLPFC. In Figure 7 it can be seen that the control group showed varying workload over each session, whereas the neuroadaptive group displayed consistent workload measures from sessions 1–3 and an increase only in session 4. *Post-hoc* tests revealed that control and neuroadaptive groups had a significant difference in measured oxyhemoglobin, and therefore mental workload, during this session. We can deduce that this increase in workload for the neuroadaptive group was due to the increase in training level. However, despite the harder difficulty, these participants displayed improved performance as compared to control. This suggests that the prior training sessions better prepared these participants for the jump in difficulty.

This pattern of consistent workload over sessions leading to faster progression and higher training levels achieved is one of the main goals of neuroadaptive training. By challenging each person at the optimal difficulty for their skill, their engagement and workload will hit the peak of the Yerkes-Dodson curve, opening the door for enhanced learning. The increased workload during session 4 proves that a certain level of mastery was attained because performance was able to match training progression.

The situation awareness task had level progression for control and neuroadaptive groups similar to the landing task, with neuroadaptive participants reaching significantly higher training levels by session 4 (Figure 8). Although no significance was found in task performance as measured by percentage of questions answered correctly after each 90 second trial, it can be seen that the control group fell after session 1 and remained at around 70%, whereas the neuroadaptive group improved in sessions 3 and 4 (Figure 10). Taking into consideration that the neuroadaptive group reached higher difficulty levels during session 4 with higher performance, we can deduce that the training method had some positive effect.

The fNIRS workload measures for situation awareness differ from the previous task (Figure 11). In optode 9 located in the right medial DLPFC, the control group shows a slight U-shaped pattern, whereas the neuroadaptive group significantly increases with each session. It is possible that due to the training level also increasing each session, and performance also improving by the end of the experimental period, that the neuroadaptive group was more engaged with the task than the control group, performing better despite the higher difficulty level. The control group did display relatively consistent workload over sessions without as much progression or performance improvement, suggesting that their training process was slower than it could have been.

The ring task results display a different pattern of progression which requires a separate interpretation. This follows from both groups significantly increasing in training level over time until session 4, where the control group reaches a higher level while the neuroadaptive group plateaus (Figure 12). The main factors of group and session showed a significant interaction, confirming that the plateau shape was distinct from the linear increase.

Subjective workload for both groups as measured by the NASA-TLX after each set of training tasks decreased over each session, but only the neuroadaptive group had significant decrease in perceived workload (Figure 13). This was due to a slightly higher perceived workload at the start, but no significant group differences were found. However, by session 3 both groups had almost identical perceived workload, suggesting that the neuroadaptive group improved more over the four training sessions than the control group.

Both measures of behavioral performance for the ring task showed similar findings (Figure 14). The ratio of rings flown through each trial started higher in the control group, significantly increasing in session 3, but decreasing in session 4. In the neuroadaptive group, performance stayed consistent until session 4, where it significantly increased as compared to session 1. Taking into consideration the training level achieved by this final session, it is clear that the control group was raised to a level above their ability, thus negatively impacting their performance in session 4. In contrast, the neuroadaptive group was able to improve their performance in session 4 because they remained at their ideal training level. A complimentary result is seen in the flight stability measure, with both groups improving over the first three sessions, but only the neuroadaptive group continuing to improve in session 4 compared to the control group which remained the same. Considering the significant interaction between group and session for ratio of rings flown through, we can confidently state that the control group level progression was not as conducive to learning as the neuroadaptive group.

The fNIRS workload measurements displayed significant interactions between group and session. Optode 11, which is in the more medial area of the right PFC, displays similar workload levels between groups during sessions 1 and 2, after which only the neuroadaptive group shows significantly decreased workload in sessions 3 and 4 (Figure 15). This implies that the neuroadaptive group advanced to an appropriate level in session 3 for their skill and set them up for success in session 4. The significant group and level interaction also tells us that the neuroadaptive group had comparatively lower workload for the same difficulty levels as control. Using the scaffold-storage framework for skill acquisition (Petersen et al., 1998), one way of interpreting this data is that optode 11 is associated with long-term skill acquisition and intrinsic load in this task, decreasing when learning is faster and increasing when learning is slower.

### Limitations and Future Considerations

In this study, we utilized optical brain imaging on the prefrontal cortex, which is known to be involved in higher cognitive processes such as working memory, attention, and executive function. Our goal for determining a generalized location from which to measure cognitive workload was decided *a priori* based on previous literature, and selected as the left dorsolateral prefrontal cortex. This method was beneficial in that analysis of workload between sessions to determine the next training level for the neuroadaptive group could be done immediately; however, our findings indicated that each task also had task-specific hemodynamic correlations with workload. These results can inform more effective neuroadaptive training in the future, and also help to refine the search for truly generalizable cognitive workload measures.

Regarding the specific flight simulator tasks used, design limitations and prior participants experience may have influenced the results. We utilized a low fidelity flight simulator with two hand controllers for participants that have no experience piloting. Initial differences in understanding the mechanics of flight, manual dexterity, and video game experience could have caused shifts in starting ability and ease of learning a novel skill, but were not recorded beforehand. The experimental design of the three tasks used and the necessity of creating a wide range of difficulty levels may have also exacerbated the difficulty of skill acquisition over a relatively short, four-session study. For example, in the landing task which required two-handed coordination of unfamiliar controls, the ability to alter speeds and turn the plane was limited in earlier levels as a form of autopilot. As training levels increased, these controls were returned to the participants. However, this could be considered as altering the task itself, even as the goal of landing and flying smoothly remained the same. The method of presenting limited segments of a complex task is known as part-task training (Wickens et al., 2013), although in this case elements were cumulatively added rather than trained separately. Training for this study was expediated as compared to a more formal learning environment, so it is possible that the full benefits of part-task training were not seen here.

Also important to consider is the number of total difficulty levels for each task, which ranged from seven to nine. If participants improved each session on a task but only went up a single level, the higher training levels would never be reached in our limited time. Thus, we designed both the control and neuroadaptive progression flowcharts to allow for level skipping in the case of exemplary performance or optimal workload. Unfortunately, the skipping of levels may have had unintended effects on learning, as new aspects of the controls may have been introduced before previous ones are learned. We accepted this concession in the experimental design to identify potential contrast between control and neuroadaptive groups, but ideally we would extend the length of the experiment to avoid these concerns in the future.

Some performance measures were challenging to obtain due to task limitations and the difficulty of flying without prior experience. Situation awareness was graded based on questions correctly answered, but on early levels with only three questions the difference between zero or one wrong answer is 33%, which has a large effect on final score. In the landing task, actually landing a plane without crashing or damaging the aircraft at higher levels when autopilot is not engaged is very difficult, making measures of true success unreliable. All participants were informed to land within certain parameters of speed and location, but very few were able to. The virtual plane used during the landing task was also very different than the one used in the ring task. In the ring task a fighter jet with high sensitivity to the controls was used in order to allow participants to fly the challenging ring course, but the change in controls between tasks may have added confusion or made learning more difficult than focusing on a single plane. Improvements to the tasks itself in the future could allow for more cohesive training overall, as well as simply using only a single task in one experiment.

### Generalization of the Approach— Suggestions of Next Steps

In this study, we utilized flight simulator tasks that we have used before. However, the neuroadaptive training approach described here could be generalized and applied for other types of tasks in different domains. In this section, we outline guidelines and suggestions for applying this neuroadaptive approach.

#### Selection of Target Brain Area, Neuroimaging Modality, and Psychophysiological Signals

The core nature of neuroadaptive training stems from the input of mental effort during task execution. For the selected task domain and type, the relevant brain areas (target regions) that are responsive to task difficulty should be identified before the training, and used as input for the adaptation algorithm. In our study, we had multiple previous experiments with similar tasks. If prior data does not exist, a separate session could be run where multiple difficulty levels of the same tasks are presented in a pseudo-random order, and the significant main effects of task difficulty for brain areas could be ascertained.

In this study, we utilized the fNIRS brain imaging modality for localized brain activity monitoring, but incorporating other brain monitoring modalities such as EEG, and psychophysiological metrics such as eye-tracking, heart rate, and respiration rate as a multi-modal approach into the algorithm could help improve efficacy.

#### Selection of Target Biomarker for Feature Extraction

Another important factor to consider is the feature extraction from the selected biosignals. In this study, we utilized slope from oxygenated-hemoglobin changes of fNIRS data; however, there are many other potential features such as range, mean, min, max, and variability that could be extracted.

#### Level Adaptation Range

In this pilot evaluation we maintained a balanced adaptation between the neuro and control groups by limiting the possible level change from −1 to +2. However, a more flexible and wider range (depending on the available levels and task difficulty) could provide more precise personalization and help improve outcomes.

Moreover, in the behavioral performance measures, different percentage ranges/threshold regimes could be implemented depending on the tasks and performance metrics used. Here we used 10–20 percent bands at the highest and lowest ends of performance. It may be beneficial to further striate these bands to incorporate a larger range of performance.

## Conclusion

In this study, we developed a novel neuroadaptive training that applies difficulty adjustment by incorporating neural correlates of fNIRS-derived workload for enhancing the evaluation of participant state during learning of complex skills. We created an adaptation algorithm that allowed for the direct comparison of a control group who progressed based on performance and a neuroadaptive group that progressed based on both performance and mental workload measures. Using a low fidelity flight simulator and three distinct, complex tasks requiring skills that most participants would not be familiar with, we found that our neuroadaptive training provided benefits over the control condition. In the landing task, neuroadaptive participants reached higher levels and displayed improved performance over control participants, and were able to engage the more difficult levels with, respectively, higher prefrontal activation. In the situation awareness task, neuroadaptive participants again reached higher levels by the end of training, also displaying better performance and mental workload that kept up with skill acquisition. In the ring task, we saw the effects of progressing too quickly, as control participants reached a higher difficulty level in mid-training, but suffered for it in both performance and mental workload at the end of training, as compared to the steadily increasing neuroadaptive group. These three sets of results show multiple applications of our fNIRS-based neuroadaptive training, and demonstrate the effectiveness in enhancing the learning of new skills. The application of this paradigm has the potential to enhance the training of complex tasks beyond piloting into the realms of surgery, teleoperation of precision machinery, air traffic control, teaching, and more.

## Data Availability Statement

The raw data supporting the conclusions of this article will be made available by the authors, without undue reservation.

## Ethics Statement

The studies involving human participants were reviewed and approved by Drexel University Institutional Review Board (IRB). The patients/participants provided their written informed consent to participate in this study.

## Author Contributions

JM performed the experiment, collected the data, analyzed the data, and prepared and wrote the manuscript. AK helped with experiment scenario implementation and setup, and participated in manuscript revision. MZ supported the idea, helped with the design of the experiment, discussed and interpreted the results, and revised the manuscript. HA initiated and supervised the study, designed the experiment, analyzed the data, discussed and interpreted the results as well as prepared, and revised the manuscript. All authors contributed to the article and approved the submitted version.

## Funding

This research was supported in part by the Lockheed Martin Advanced Technology Laboratories Award Number 4102647914.

## Author Disclaimer

The content of the information herein does not necessarily reflect the position or the policy of the sponsor and no official endorsement should be inferred.

## Conflict of Interest

fNIR Devices, LLC manufactures the optical brain imaging instrument and licensed IP and know-how from Drexel University. HA was involved in the technology development and thus offered a minor share in the startup firm fNIR Devices, LLC. AK and MZ were employed by Lockheed Martin. The remaining author declares that the research was conducted in the absence of any commercial or financial relationships that could be construed as a potential conflict of interest.

## Publisher's Note

All claims expressed in this article are solely those of the authors and do not necessarily represent those of their affiliated organizations, or those of the publisher, the editors and the reviewers. Any product that may be evaluated in this article, or claim that may be made by its manufacturer, is not guaranteed or endorsed by the publisher.
