# Supplementary material for: Neuroadaptive Training via fNIRS in Flight Simulators
Source: Front Neuroergon. 2022 Mar 30;3:820523. doi: 10.3389/fnrgo.2022.820523 (PMC10790906; doi:10.3389/fnrgo.2022.820523)
Supplement: Supplementary file 1 [file Data_Sheet_1.pdf]

Supplementary Material

Neuroadaptive Training via fNIRS in Flight Simulators

Statistical results for all fNIRS HbO optodes are included in the tables below.

fNIRS Group Main Effect

	Landing		Ring		Situation Awareness	
**Optode**	**F**	**p**	**F**	**p**	**F**	**p**
**1**	1.52	0.2241	0.16	0.6877	4.48	0.0369
**2**	2.21	0.1428	0.00	0.9612	4.32	0.0406
**3**	0.02	0.8854	0.09	0.7683	1.57	0.2138
**4**	1.53	0.2190	0.02	0.8880	2.10	0.1514
**5**	0.18	0.6687	1.21	0.2779	4.22	0.0426
**6**	0.36	0.5506	1.84	0.1810	2.90	0.0920
**7**	0.94	0.3378	2.02	0.1647	0.17	0.6827
**8**	0.01	0.9308	2.47	0.1252	0.07	0.7868
**9**	0.05	0.8288	6.64	0.0146	9.30	0.0030
**10**	0.14	0.7057	6.81	0.0129	8.08	0.0055
**11**	0.00	0.9763	10.33	0.0034	0.01	0.9043
**12**	0.07	0.7951	7.96	0.0085	0.01	0.9026
**13**	0.92	0.3393	1.18	0.2812	4.59	0.0352
**14**	0.54	0.4621	1.37	0.2453	3.90	0.0516
**15**	0.85	0.3613	0.31	0.5825	0.80	0.3741
**16**	0.55	0.4588	0.50	0.4824	0.69	0.4091

Table S1: fNIRS HbO significance values for group main effect from generalized linear models using group and session as fixed effects, level as covariate, and subject as random factor.

fNIRS Session Main Effect

	Landing		Ring		Situation Awareness	
**Optode**	**F**	**p**	**F**	**p**	**F**	**p**
**1**	0.58	0.6245	5.86	0.0007	15.50	0.0000
**2**	2.04	0.1095	5.47	0.0013	12.11	0.0000
**3**	0.44	0.7206	11.95	0.0000	15.15	0.0000
**4**	3.03	0.0309	11.31	0.0000	13.49	0.0000
**5**	0.76	0.5181	11.72	0.0000	17.37	0.0000
**6**	1.86	0.1368	9.75	0.0000	13.19	0.0000
**7**	0.72	0.5385	6.91	0.0002	10.15	0.0000
**8**	4.06	0.0080	6.11	0.0006	8.21	0.0000
**9**	1.12	0.3414	24.53	0.0000	13.98	0.0000
**10**	1.19	0.3143	21.07	0.0000	10.42	0.0000
**11**	0.94	0.4191	14.52	0.0000	6.08	0.0006
**12**	3.01	0.0315	13.70	0.0000	5.21	0.0018
**13**	1.34	0.2616	5.33	0.0015	5.09	0.0021
**14**	1.31	0.2727	4.93	0.0026	3.34	0.0207
**15**	1.63	0.1839	7.68	0.0001	9.60	0.0000
**16**	4.73	0.0034	5.47	0.0013	5.99	0.0007

Table S2: fNIRS HbO significance values for session main effect from generalized linear models using group and session as fixed effects, level as covariate, and subject as random factor.

fNIRS Group * Session Interaction

	Landing		Ring		Situation Awareness	
**Optode**	**F**	**p**	**F**	**p**	**F**	**p**
**1**	1.72	0.1649	0.79	0.5017	6.19	0.0005
**2**	1.59	0.1929	0.59	0.6196	4.65	0.0038
**3**	1.27	0.2871	3.28	0.0225	3.47	0.0176
**4**	1.09	0.3522	2.22	0.0883	3.17	0.0257
**5**	2.55	0.0576	2.74	0.0450	4.29	0.0061
**6**	1.71	0.1669	2.69	0.0480	2.57	0.0559
**7**	1.44	0.2329	0.33	0.8021	5.39	0.0014
**8**	1.12	0.3403	0.34	0.7958	4.21	0.0067
**9**	1.90	0.1313	1.29	0.2772	11.08	0.0000
**10**	1.82	0.1443	1.43	0.2360	8.88	0.0000
**11**	1.04	0.3749	3.33	0.0210	3.43	0.0183
**12**	0.36	0.7794	2.56	0.0572	2.72	0.0460
**13**	4.29	0.0061	0.77	0.5095	9.17	0.0000
**14**	3.79	0.0114	0.88	0.4525	7.32	0.0001
**15**	0.13	0.9429	0.81	0.4859	10.93	0.0000
**16**	0.39	0.7568	0.86	0.4636	7.35	0.0001

Table S3: fNIRS HbO significance values for group*session interaction from generalized linear models using group and session as fixed effects, level as covariate, and subject as random factor.
